# A Method for Removal of Low Frequency Components Associated with Head Movements from Dual-Axis Swallowing Accelerometry Signals

**DOI:** 10.1371/journal.pone.0033464

**Published:** 2012-03-29

**Authors:** Ervin Sejdić, Catriona M. Steele, Tom Chau

**Affiliations:** 1 Department of Electrical and Computer Engineering, University of Pittsburgh, Pittsburgh, Pennsylvania, United States of America; 2 Toronto Rehabilitation Institute and Department of Speech-Language Pathology, University of Toronto, Toronto, Ontario, Canada; 3 Bloorview Research Institute, Holland Bloorview Kids Rehabilitation Hospital and Institute of Biomaterials and Biomedical Engineering, University of Toronto, Toronto, Ontario, Canada; Emory University/Georgia Insititute of Technology, United States of America

## Abstract

Head movements can greatly affect swallowing accelerometry signals. In this paper, we implement a spline-based approach to remove low frequency components associated with these motions. Our approach was tested using both synthetic and real data. Synthetic signals were used to perform a comparative analysis of the spline-based approach with other similar techniques. Real data, obtained data from 408 healthy participants during various swallowing tasks, was used to analyze the processing accuracy with and without the spline-based head motions removal scheme. Specifically, we analyzed the segmentation accuracy and the effects of the scheme on statistical properties of these signals, as measured by the scaling analysis. The results of the numerical analysis showed that the spline-based technique achieves a superior performance in comparison to other existing techniques. Additionally, when applied to real data, we improved the accuracy of the segmentation process by achieving a 27% drop in the number of false negatives and a 30% drop in the number of false positives. Furthermore, the anthropometric trends in the statistical properties of these signals remained unaltered as shown by the scaling analysis, but the strength of statistical persistence was significantly reduced. These results clearly indicate that any future medical devices based on swallowing accelerometry signals should remove head motions from these signals in order to increase segmentation accuracy.

## Introduction

Patients living with the effects of stroke or neurodegenerative conditions commonly encounter swallowing difficulties (dysphagia) [1]. Dysphagia occurs for various reasons in these patients (e.g., damage to the cranial nerves associated with the swallowing neural control centers) [2] [Bibr pone.0033464-Reddy2]–[4]. These patients have an increased risk for aspiration (the entry of material into the airway below the true vocal folds), which may cause asphyxiation and other severe consequences [5], [6]. The videofluoroscopic swallowing study (VFSS) is the current gold standard for detection and management of dysphagia [7]. Nevertheless, the VFSS is not suitable for ongoing monitoring for various reasons including excessive exposure to radiation, long waiting lists at hospitals and unavailability of the equipment at every hospital [8], [9]. In recent years, swallowing accelerometry has emerged as an alternative approach for the non-invasive assessment of swallowing function (e.g., [2], [3]) and it involves the measurement of epidermal vibrations using an accelerometer on the patient's neck. Due to the presence of two-dimensional movement of the hyoid and the larynx during swallowing [10], [11], dual-axis accelerometers provide more accurate results [12], [13] than single-axis accelerometers [14] [Bibr pone.0033464-Chau1] [Bibr pone.0033464-Das1]–[17].

Previous contributions have observed that swallowing accelerometry signals contain low frequency components associated with head motions (e.g., [12], [13]). Extensive head motions can severely alter the amplitudes of dual-axis swallowing accelerometry signals. As a consequence, the accuracy of segmentation process can be affected by these head motions [12]. Similarly, these movements also affect the statistical properties of signals (e.g. [18], [19]). Therefore, there is a growing need for the automatic removal of these low frequency components associated with head motions from swallowing accelerometry signals. The goal of this paper is to develop such a system in order to increase the accuracy of processing steps of these dual-axis swallowing signals (e.g., improved segmentation of swallowing signals).

## Methods

### Data collection

**Figure 1:**
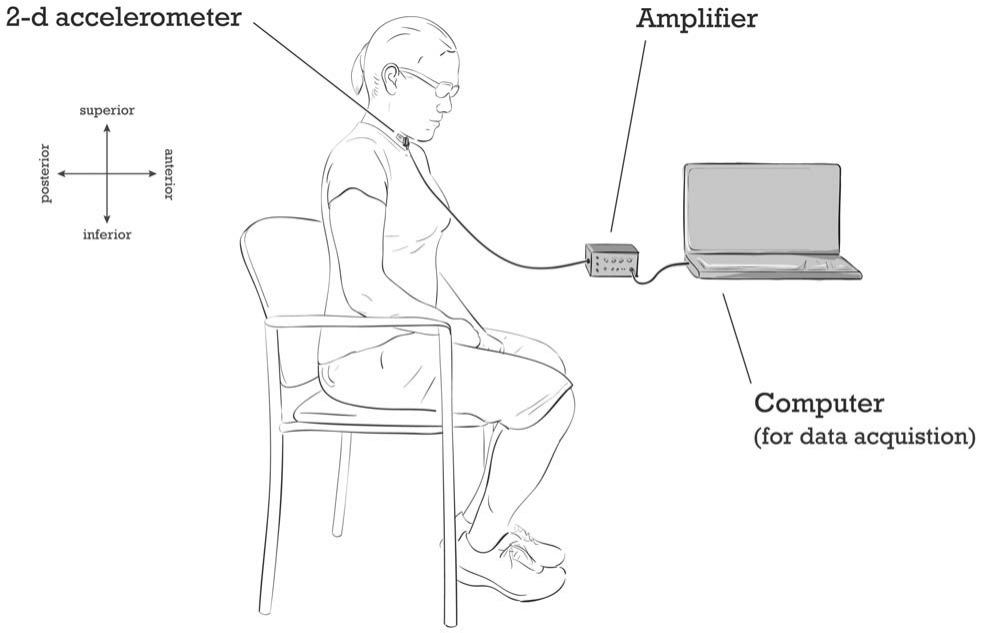
A dual-axis accelerometer attached to the participant's neck.

In this paper, we analyzed the data collected in previous studies of swallowing accelerometry [12], [20], [21], [22]. The reader should refer to those publications for full details regarding the experiment. Here, we only provide the most essential details. We recruited four hundred and eight (408) participants (aged 18–65) who provided written consent and had no known prior swallowing disorders. The study protocol was approved by the research ethics boards of the Toronto Rehabilitation Institute and Holland Bloorview Kids Rehabilitation Hospital, both located in Toronto, Ontario, Canada. A dual-axis accelerometer (ADXL322, Analog Devices) was attached to the participant's neck (anterior to the cricoid cartilage) using double-sided tape. The axes of acceleration were aligned to the anterior-posterior and superior-inferior directions, as shown in Figure 1. Data were band-pass filtered in hardware with a pass band of 0.1–3000 Hz and sampled at 10 kHz using a custom LabVIEW program running on a laptop computer. Data were saved for subsequent off-line analysis.

First, each participant was cued to perform 5 saliva swallows. Subsequently, the participant completed 5 water swallows by cup with their chin in the natural position (i.e., perpendicular to the floor) and 5 water swallows in the chin-tucked position (i.e., as depicted in Figure 1, with the instruction to look down at the knees while swallowing). The entire data collection session lasted 15 minutes per participant.

### Splines and dual-axis swallowing accelerometry signals

A typical swallowing accelerometry signal, 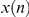, can be expressed as:
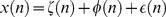
(1)where 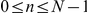 and 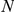 represents the length of the signal, 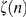 is a low-frequency signal associated with head motions, 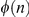 is a signal associated with swallowing activities of a person, and 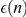 is the additive white Gaussian noise with variance 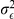. As pointed out in previous contributions (e.g., [12], [13]), any head motion can significantly alter the amplitudes of dual-axis swallowing accelerometry signals and hence confound any consequent data processing steps. Therefore, we need to diminish the effects of 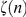, while not removing any parts of 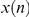 associated with swallowing, i.e., 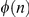. A possible approach to achieve this goal is to obtain samples of 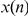 using lower sampling frequencies (i.e., to resample 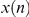 using lower sampling frequencies) in order to extract 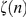 while neglecting components associated with 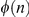 and 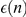. In order to accomplish this goal we implement splines [23], [24], [25], as they provide the best cost-performance trade-off among all interpolation/approximation methods [26].

In order to gain a better understanding of our approach, we outline the basic properties of splines in “Spline interpolations” followed by an explanation how splines can be used for approximation. Then, in “Splines as a tool for removal signal components associated with head movements” we describe how splines can be used for removal of low frequency components associated with head movements.

#### Spline interpolations

In simple words, splines of order 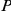 are equal to polynomials of degree 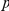 on each interval, and subsequent intervals are smoothly connected together [24], [25]. The connecting points are called knots [23], [24], [25]. It should be pointed out that the polynomials are connected in such a way that the overall function is 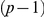 times continuously differentiable even at the knots [23], [24], [25]. One of the first contributions in the field showed that discrete samples of a continuous signal, 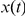, can be represented using a 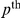 order spline with equidistant knots using the following equation [23], [24], [25]:
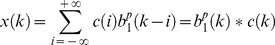
(2)where 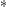 represents convolution, 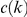 is an 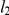 sequence of real numbers and 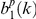 is the finite impulse response of the operator known as the indirect spline filter of order 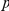, i.e. the discrete B-spline. In other words, for 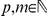, the discrete B-spline, 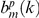, is defined as a sequence of integral samples of the corresponding 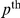 order continuous B-spline, 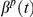, expanded by a factor of 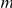 [23], [24], [25]:
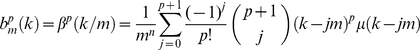
(3)where
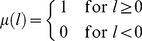
(4)and
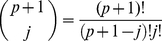
(5)with the following starting condition:
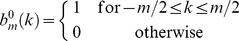
(6)


The B-splines of order 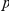 are compactly supported and symmetric around zero [23], [25], [27]. Furthermore, they provide a basis of the subspace of all continuous piecewise polynomial functions of degree 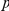 with derivatives up to order 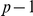 that are continuous everywhere on the real line [23], [25], [27]. The zeroth order discrete spline, 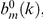 is a rectangular window of width 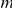 that is centered with respect to the origin when 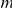 is odd. This operator corresponds to a moving average filter of size 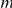 that can be implemented recursively using a standard update procedure (two operations per sample value). For discrete B-splines with upsampling integer 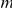 greater than 1, a convolution property can be established [23], [24], [25]:

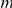
 is odd:
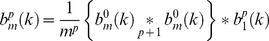
(7)

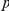
 odd and 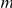 even:
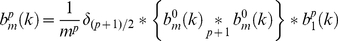
(8)where 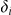 is a shift operator;

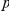
 even and 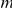 even:
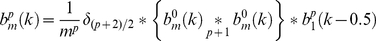
(9)

where 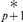 denotes 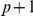 consecutive convolutions of a signal with itself. These equations demonstrate that discrete B-splines of various widths can be constructed from repeated convolution of simple moving average filters (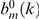) and a correction kernel (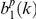). The main advantage is that both the direct spline transform (the process of determining the expansion coefficients) and the indirect spline transform (the process of reconstructing the original sampled values with an optional interpolation) can be interpreted as simple filtering operations. Therefore, the above result suggests that that the spline coefficients, 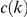, can be determined simply by inverse filtering [23], [25], [27]. Also, in the case that one desires to obtain the discrete signal, 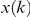, at a higher sampling rate, i.e., using an integral up-sampling factor 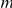, then the reconstruction given by (2) can be rewritten as [23], [25], [27]:
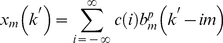
(10)Furthermore, if a basic operation of the up-sampling of a signal 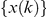 by a factor 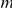 which produces the new sequence 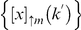 defined as
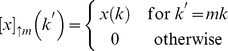
(11)then by substituting 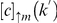 in (10), it can be rewritten as [25], [27]:
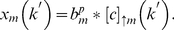
(12)It should be pointed out that the requirement of 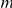 being an integer is not a major limitation since any rational sampling rate can be obtained from a succession of integral interpolations and decimations [25], [27].

#### Spline approximations

The use of B-splines extends beyond simple interpolation. In particular, we are interested in obtaining B-spline approximations [27]. They are quite useful in noise reduction and data compression (e.g., [28]). In our case, we use them to approximate low frequency components associated with head movements, which are present in the dual-axis swallowing accelerometry signals. Such B-splines approximations can be obtained by imposing smoothness constraints on the solution (smoothing splines), or by reducing the number of coefficients (least square approximation) [24], [29]. Nevertheless, the smoothing spline has as many coefficients as the initial signal, which is not desired in our case due to computational complexity. It has been suggested that by implementing the so-called least square splines we can deal with fewer degrees of freedom [24]. De Boor described a general method for determining such solutions in the case of arbitrarily spaced data points that relies on the use of standard least squares approximation techniques [24]. The approach used here considers equally spaced nodes since it leads to substantial computational simplifications. When dealing with discrete signals, this approximation method involves some form of decimation of the spline coefficients [24], [29]. This technique is conceptually similar to resampling a signal at a lower rate which requires the use of an antialiasing filter for bandlimited approximation of a signal with minimum error. In this sense, the present theory of B-splines fitting is an extension of the conventional sampling theorem for the subspace of piecewise polynomial functions of class 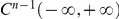 with equally spaced nodes [24], [29].

In our case, we wish to determine the least squares spline coefficients that minimize the approximation error:
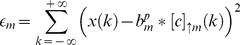
(13)and it has been shown that the expression can be solved by inverse filtering [25], [27]:
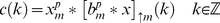
(14)where the postfilter 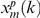 is defined by
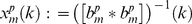
(15)These results suggest a simple three step procedure for the determination of the least square B-spline coefficients [29]. First, we perform a prefiltering with a B-spline kernel of width 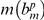. This operation is equivalent to an indirect B-spline transform. Second, a decimation by a factor of 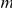 is carried out. This step samples the signal at the position of the knots of the expanded B-spline basis functions. Third, we conduct a postfiltering of a decimated sequence with the least squares operator.

#### Splines as a tool for removal signal components associated with head movements

Our approach is based on the idea of approximating 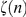 and removing it from 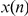. However, since we only have access to 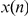, our algorithm approximates 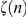 using the least squares spline approximation of 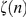 and removes it from 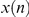 with following steps:

- Using 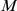 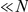 knots, determine a least square approximation of the expansion coefficients using
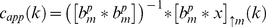
(16)
- Using the approximated expansion coefficients, reconstruct 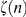 using the B-spline indirect transformations:
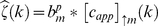
(17)
- We remove the low frequency components associated with head movements using 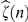:
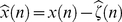
(18)

An important question here is how do we choose 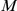
? First, let's consider the meaning of 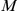. Essentially, 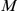 represents the number of knots, that is, the number of samples that we will use to approximate these low frequency components. Hence, M is proportional to the number of samples, N, and to this new (re)sampling frequency. The frequency has to be sufficiently small in order to avoid sampling vibrations associated with swallowing. On the hand, it has to be sufficiently high to appropriately sample the low frequency vibrations associated with the head movements. The question is how do we choose this new (re)sampling frequency? There is no fundamental difference between the process of performing a least-square spline approximation of a signal and obtaining its band-limited representation using the standard sampling procedure dictated by Shannon's theory [25]. However, is there any equivalent of the sampling theorem that tells us that the signal can be reconstructed exactly if it is sampled at a frequency 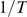 that is at least twice the Nyquist rate? In principle, one should expect a similar result, at least for higher-order splines [25]. Because we are performing an orthogonal projection, the approximation error will be generally non-zero unless the signal is already included in the approximation space [25]. However, we can hope to control this error by choosing a sampling step 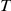 that is sufficiently small. To analyze this situation, which is more complicated that the traditional band-limited case, we turn to approximation theory. A fundamental result is that that the rate of decay 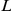 of the error as a function of T depends on the ability of the representation to reproduce polynomials of degree 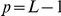. The approximation error also depends on the bandwidth of the signal [25]. The relevant measure in this context is [25]:
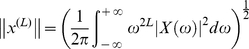
(19)where 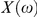 denotes the Fourier transform of 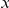; this is nothing but the norm of the 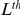 derivative of 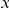. The key result from the Strang-Fix theory of approximation is the following error bound [25]:
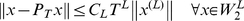
(20)where 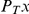 is the least-squares spline approximation of 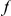 at sampling step 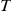 and 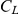 is a known constant. 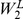 denotes the space of functions that are 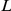 times differentiable in the finite-energy sense. In other words, the error will decay like 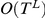, where the order 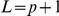 is one more than the degree 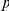 [25].

Hence, using these previous findings, we used 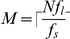, where 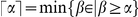, 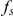 is the original sampling frequency and 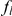 is the lower sampling frequency. Specifically, this 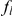 is proportional to the frequency associate with head motions. A recent contribution showed that the frequencies associated with head motions are given by the following interval 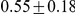 Hz in the A-P direction, and 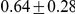 Hz in the S-I direction [30]. Therefore, we expect that 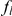 is at least twice the highest frequency in each direction. However, a numerical analysis, as described in Section “Data analysis”, will be carried out to determine a precise value of 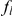 for the A-P and S-I directions.

Due to the fact that the approximation error decreases proportionally to the order of the approximating polynomial, we used fourth order splines in our research. Specifically, we choose a fourth order, since it has been shown that there are no significant gains in performance between the fourth and higher order polynomials (e.g., fifth and sixth orders), while schemes based on higher order polynomials take longer time to execute [26].

### Data analysis

Our data analysis was a three-part process. The first two parts involved the analysis of the proposed approach using synthetic signals, while the third part involved the analysis of real dual-axis swallowing accelerometry signals that were acquired as described in Section “Data collection”.

In the first part, we simulated swallowing signals to analyze the effects of varying signal-to-noise ratio (SNR) on the value of 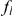. Our goal was to systematically analyze whether or not statistically equivalent values of 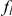 were obtained under various noise conditions. To ensure that the test signals mimicked the dual-axis swallowing accelerometry signals (i.e. the signal model depicted by eqn. (1)), we split the modeling into two parts. The model for 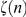 is given by:
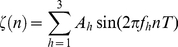
(21)where 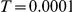 seconds; 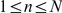 and 
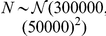
 with a constraint that 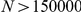. 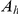 is uniformly drawn from 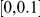 for the A-P direction and from 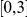 for the S-I direction. Similarly, 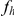 is uniformly drawn from 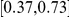 Hz for the vibrations in the A-P direction, and from 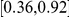 Hz for the vibrations in the S-I direction, where these frequency bands were based on the results of a spectral analysis of data from [30]. To model the combined effects of 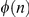 and 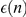, we implemented a model used in [12], which mandated that there should be five distinct intervals where the variance of the signals increase above the baseline variance and each of the five intervals should have random duration and random frequency components to mimic intersubject variations. 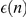 was represented by additive white Gaussian noise with variance 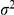. The synthetic swallowing signals are given by:
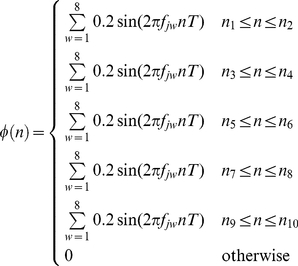
(22)where 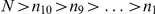; 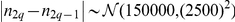 for 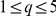 with a constraint that 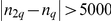; 
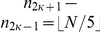
 where 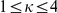; and 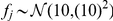. It should be noted that the models for low-frequency components associated with head motions given by eqn. (21) and dual-axis swallowing accelerometry signals given by eqn. (22) do not necessarily represent realistic signals, since the physiological characteristics of such signals are still largely unknown. The proposed model rather depicts statistical behaviour of those signals, i.e. the activity regions have higher variances than the baseline regions, and is only used for an accuracy analysis of the proposed algorithm. Also, for the purpose of notational simplicity we only discuss vibrations in one direction. Nevertheless, these equations apply to both directions.

Using these simulated signals, our goal was to investigate the optimal value of 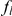 in each direction, i.e., the value of 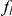 providing us with the smallest error. Specifically, we varied the value of SNR between 0 dB and 30 dB in 5-dB increments and calculated the mean square error (MSE) using 100 realizations of
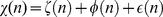
(23)as follows:
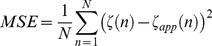
(24)where 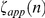 represents the approximated low frequency oscillations.

In the second part of the analysis we compared performances of our approach against well known approaches based on empirical mode decomposition (EMD) [31], the smoothness priors method (SPM) [32], and piecewise polynomial fitting (PPF) [33]. Specifically, for SPM we divided signals into 1000 subintervals, while for PPF signals were divided into 5000 subintervals. Also, we used second-order polynomials for PPF. We compared these four methods for various levels of SNR ranging from 0 to 30 dB in 1 dB increments. For each SNR value, we calculated the normalized MSE (NMSE) as:
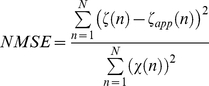
(25)NMSE was calculated using 500 realizations of (23) under two different conditions. One condition implied that we generated new versions of 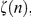 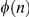, and 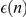 with each new realization of (23). The second condition involved keeping 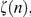 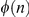 constant, while obtaining a new version of 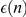 for each new realization.

The third part of our experiment involved the analysis of real swallowing signals as described in Section “Data collection”. Specifically, we examined the effect of removing low frequency components associated with head movements on segmentation accuracy and statistical persistence observed in these signals. To investigate the effects on the segmentation accuracy, we initially segmented recordings containing head movements using the procedure described in [12]. For each recording, we denoted the number of swallows present, the number of correctly segmented swallows (CSS), the number of false positives (NFP) and the number of false negatives (NFN). A swallow was considered correctly identified only when more than 90% of the swallow duration was captured by the segmentation process. As the second step of this analysis, the recordings were pre-processed using the proposed approach and segmented as outlined in [12]. CSS, NFP and NFN were subsequently computed and compared to values obtained without head movement removal. To investigate the effects of head movement removal on statistical persistence, our last step of data analysis involved detrended fluctuation analysis (DFA) of dual-axis swallowing accelerometry signals. Specifically, we followed the steps outlined in [18], [19]. Our goal was to compare the strength of the statistical persistence (reflected through the so-called scaling exponent, 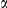) before and after removal of low frequency components. In other words, we examined whether previously reported trends in statistical persistence were observed even when we removed these components.

In order to establish statistical significance of our results, a non-parametric inferential statistical method known as the Mann-Whitney test was used [34], which asssses whether observed samples are drawn from a single population (i.e., the null hypothesis). For multi-group testing, the extension of the Mann-Whitney test known as the Kruskal-Wallis was used [35]. A 5% significance was used.

## Results and Discussion

Table 1 summarizes the results of the first part of the analysis. From these results, it is obvious that the sampling frequency in either direction remains equal regardless of the noise level (Kruskall-Wallis, 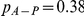, 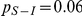). Therefore, the average sampling frequency in the A-P direction is 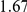 Hz, while the average sampling frequency in the S-I direction is 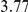 Hz. These average values will be used in further analysis of the simulated and real data. As described in Section “Splines as a tool for removal signal components associated with head movements”, it is rather clear that we can find an approximate value of the lower sampling frequency that will be consistent in terms of the error.

**Table 1:** Values of 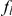 in both directions providing the smallest MSE.

	0 db	5 dB	10 dB	15 dB	20 dB	25 dB	30 dB
A-P	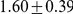	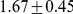	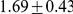	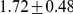	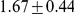	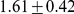	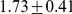
S-I	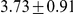	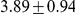	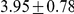	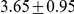	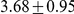	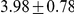	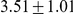

**Figure 2:**
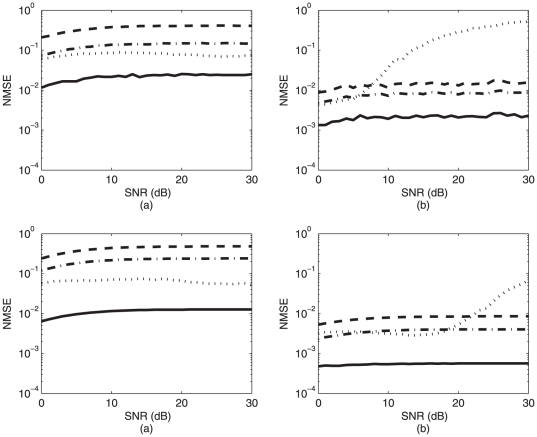
A comparison of accuracies for the proposed method (solid line), SPM (dashed line), PPF (dash-dotted line) and EMD (dotted line). (a) and (b) represent NMSE in the A-P and S-I directions, respectively, while generating new versions of 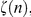 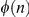, and 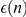 with each new realization of eqn. (23). (c) and (d) represent NMSE in the A-P and S-I directions, respectively, while keeping 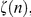 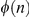 constant and obtaining a new version of 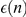 for each new realization of eqn. (23).

Figure 2 depicts the results of the comparison analysis. The proposed method (solid line) achieves the smallest MSE regardless of the condition, followed by the EMD approach in the A-P direction and the PPF approach in the S-I direction. Except with the EMD approach, all other methods exhibited consistent error across various values of SNR. This is a desirable property in real-life applications, since it does not necessitate apriori denoising of signals. It should be mentioned that EMD can produce larger errors than other methods as shown in Figure 2. This is mainly due to the fact that EMD cannot differentiate properly between low frequency oscillations associated with the trend and higher frequency oscillations associated with the swallowing phenomenon.

Table 2 represents the results of the segmentation process accuracy. In particular, these results clearly depict that removing low frequency components associated with head movement increases accuracy. Overall, we witness a 27% drop in the number of false negatives and a 30% drop in the number of false positives.

**Table 2:** Segmentation accuracy before and after removing low-frequency components.

		Before removal			After removal		
Swallowing type	TNS	CSS	NFP	NFN	CSS	NFP	NFN
Dry swallows	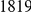	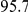	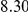	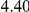	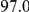	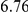	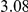
Wet swallows	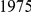	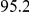	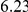	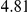	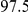	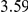	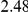
Wet chin tuck	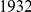	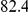	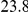	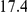	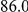	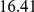	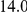
Overall	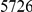	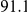	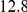	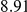	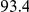	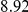	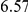

TNS = total number of swallows; CSS = correctly segmented swallows; NFP = number of false positives; NFN = number of false negatives.

The results for the last part of our analysis are summarized in Table 3. From these results it is clear that statistical persistence was significantly reduced after removing low frequency components. In fact, this finding was anticipated in [19], where we suggested that the expected 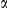 values should be between 0 and 0.5, given that segments with small fluctuations (i.e., baseline) are intermingled with segments possessing larger fluctuations (i.e., swallows). Furthermore, we anticipated that the underlying baseline characteristics (e.g., weak vibrations caused by vasomotion) and head motions were largely responsible for the observed statistical persistence. In [19], we also concluded that these temporal dependencies should be considered in the development of accelerometry-based decision support tools. The findings presented in this paper confirmed our anticipatory remarks. They clearly show that these low-frequency components should be removed since they could potentially affect the accuracy of an accelerometry-based decision support tool.

**Table 3:** Comparison of the scaling exponent, 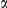, before and after low-frequency components.

	Before removal		After removal	
Swallowing type	A-P	S-I	A-P	S-I
Dry swallows	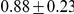	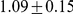	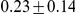	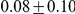
Wet swallows	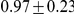	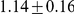	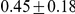	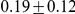
Wet chin tuck	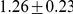	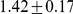	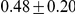	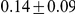

We should mention that removing low frequency components associated with head motions did introduce any previously unreported trends [19]. In particular, consistent with previous studies, there were no gender (Mann-Whitney test, 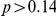), age (linear regression, 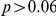) or BMI (linear regression, 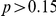) differences in either direction.

### Conclusion

In this paper, an approach for the removal of low frequency components associated with head movements was proposed for dual-axis swallowing accelerometry signals. The scheme is based on spline least square approximations of the signal and is well-suited for long signals. First, we carried out a comparative analysis of our spline-based scheme against well-known approaches using synthetic signals. We found that the spline-basesd scheme provided more accurate segmentation results. Then, dual-axis swallowing accelerometry signals collected during 3 swallowing tasks completed by 408 healthy participants were processed using the spline approach. In particular, we found that by removing low frequency components associated with head movements we increased the accuracy of the segmentation process. Nevertheless, we observed a significant decrease in the strength of statistical persistence suggesting that any accelerometry-based decision support tools should remove these low-frequency components since they could confound the decision support process.
